# The Minderoo-Monaco Commission on Plastics and Human Health

**DOI:** 10.5334/aogh.4083

**Published:** 2023-03-21

**Authors:** Editorial to accompany the Minderoo-Monaco Commission on Plastics and Human Health in the journal, Annals of Global Health, Maria Neira

**Affiliations:** 1 WHO, CH

**Keywords:** plastic life cycle, human health, microplastics, plastic additives, environmental health

The potential impact of plastics on human health has become an increasing concern, including for WHO, and most recently through the Minderoo-Monaco Commission on Plastics and Human Health.

In 2019, and following a preliminary review of microplastics in drinking water, WHO already had issued a call for research and a crackdown on plastic pollution. At the time, WHO urgently stated that more knowledge needed to be generated about the health impact of microplastics and consequently called for a stop in the rise in plastic pollution worldwide.

WHO subsequently expanded its review of this issue, including through a report released in 2022 entitled, “Dietary and inhalation exposure to nano- and microplastic particles and potential implications for human health.” While knowledge gaps still precluded a definitive assessment, a key recommendation of the report was that there should be increasing public awareness and an overwhelming consensus among all stakeholders that plastics do not belong in the environment, and measures should be taken to mitigate exposure to nano- and microplastic particles.

Of course, health concerns go beyond microplastics and relate to plastics as a whole. WHO recognizes that plastic litter can be found throughout the environment, including on streets, fields, and beaches, disturbing quality of life, and in some cases impacting tourism-related livelihoods. Poorly managed plastic waste streams can contribute to the obstruction of storm drains and sewers, which prevents proper drainage and increases sanitation-related risks. Incineration of plastic waste, with its resulting air pollution, and the climate change-inducing greenhouse gas associated with plastic production, could impact human health and the environment.

WHO recognizes that plastics play an important role in health care, but also that the health sector can and should do more to reduce plastic pollution and encourage sustainable consumption, and that doing so will promote and protect both human health and the environment, contributing to sustainable development. Waste from the health care sector requires special attention, as highlighted in a report released by WHO in 2022 entitled, “Global analysis of health care waste in the context of COVID-19.” Protective equipment such as gloves and masks, cleaning products, and single-use products all produce significant plastic waste.

With this Commission supporting the world’s adoption of a strong, comprehensive Global Plastics Treaty, it will be essential that Treaty negotiators consider protection of human health to be an essential element of any sustainable approach to address plastic pollution. It will also be important to understand that approaches to addressing plastic pollution should be modified as scientific knowledge of risk develops and that addressing this issue does not create new problems.

Identification of win-win actions are essential. To take just one example, increased reduction in tobacco consumption will also reduce the 4.5 trillion cigarette butts containing cigarette filters made of plastic that are discarded every year.

At the government level, we need a health in all polices approach. In practice, this means cooperation between sectors such as environmental protection, tobacco control, agriculture and fisheries, foreign affairs, and trade to develop health-centered regulation of plastics.

WHO, together with the health sector, will continue to take action to beat plastic pollution.

